# High-volume buprenorphine prescribers: Examining state policy contexts

**DOI:** 10.1016/j.dadr.2025.100406

**Published:** 2025-12-16

**Authors:** Megan S. Schuler, Flora Sheng, Brendan Saloner, Adam J. Gordon, Bradley D. Stein

**Affiliations:** a RAND, 1200 S Hayes St, Arlington, VA 22202, USA; b Department of Health Service, Policy, and Practice, Brown University School of Public Health, 121 S Main St, Providence, RI 02903, USA; c Department of Internal Medicine, University of Utah School of Medicine, 30 N 1900 E, Salt Lake City, UT 84132, USA; d RAND, 4570 Fifth Ave #600, Pittsburgh, PA 15213, USA.

**Keywords:** Buprenorphine, Opioids, Treatment, State policy, Mandatory behavioral health, Counseling

## Abstract

**Background:**

Most patients prescribed buprenorphine depend on a small number of high-volume prescribers. However, little is known about how state policies may affect high-volume prescribing.

**Methods:**

We used 2009–2018 IQVIA Real World Data – Longitudinal Prescriptions (a national dataset capturing approximately 90 % of U.S. retail pharmacy transactions) to examine associations between four state policies and high-volume buprenorphine prescribing (i.e., clinicians averaging ≥30 active buprenorphine patients/month). Using multivariable event-time linear probability models, we estimated the percentage-point changes in the share of prescribers qualifying as high-volume in the three years following the implementation of: (1) mandatory behavioral health counseling when prescribing buprenorphine, (2) mandatory substance use disorder-related continuing medical education (CME) for licensure, (3) Affordable Care Act (ACA) Medicaid expansion, and (4) mandatory prescription drug monitoring program (PDMP) laws.

**Results:**

Of 109,218 clinicians who prescribed buprenorphine, 8.8 % were classified as high-volume prescribers for at least one year. Both high- and low-volume prescribers increased substantially over the study period, with total prescribers rising nearly 70 %. Policies mandating behavioral health counseling were consistently significantly associated with an increase in the share of high-volume prescribers post-implementation (Y1: +3.4 %age points; Y2: +5.1 %age points; Y3: +3.2 %age points). Conversely, mandatory PDMP laws were correlated with a decreased share of high-volume prescribers (Y1: −1.6 %age points; Y2: −3.2 %age points; Y3: −4.1 %age points). No significant associations were found for mandatory CME or ACA Medicaid expansion.

**Conclusions:**

The proportion of buprenorphine prescribers who are high-volume increased during 2009–2018, reflecting faster growth among high-volume prescribers. Our findings indicate that state policies coincided with differential patterns of growth.

## Highlights

• Nationally, both high- and low-volume prescribers increased, with faster growth among high-volume prescribers.
• 8.8 % of buprenorphine prescribers qualified as high-volume in at least one year.
• Behavioral counseling policies were associated with an increase in high-volume prescribing.
• Mandatory PDMP laws were associated with a decrease in high-volume prescribing.
• No significant associations observed for CME requirements or Medicaid expansion.

## 1 Introduction

The opioid epidemic in the United States remains a critical public health crisis. Although fatal overdoses began to decline in 2023, over 87,000 individuals died from drug overdose in the U.S. from October 2023 to September 2024 (Centers for Disease Control and Prevention, 2025, Garnett and Miniño, 2024). Based on the 2023 National Survey on Drug Use and Health (NSDUH), an estimated 5.7 million Americans live with opioid use disorder (OUD) (Substance Abuse and Mental Health Services Administration, 2024). Medication treatment for opioid use disorder (MOUD), including buprenorphine and methadone, is recognized as the clinical standard of care and is associated with reduced overdose rates and improved outcomes (Golan et al., 2022, Larochelle et al., 2018). MOUD availability has expanded, yet fewer than 30 % of people with OUD receive it, indicating that a large unmet treatment need remains (Mauro et al., 2022).

Approved by the FDA in 2002, buprenorphine has become the most widely used MOUD (Substance Abuse and Mental Health Services Administration, 2024). Unlike methadone, which is dispensed only through federally regulated opioid treatment programs, buprenorphine can be prescribed in office-based settings. Until the X-waiver requirement was repealed in December 2022, clinicians needed to obtain a federal “X-waiver,” typically by completing mandated training, before prescribing buprenorphine (Saloner et al., 2021). Prior research has demonstrated that the buprenorphine prescribing landscape has been shaped by a complex interplay of state and federal policies and regulations. Key legislative and regulatory measures include the Comprehensive Addiction and Recovery Act (CARA, 2016), which allowed advanced practice providers (APPs) to obtain waivers; state laws requiring clinicians to consult prescription drug monitoring programs (PDMPs) before prescribing opioids; state-mandated continuing medical education (CME) on substance use disorders for licensure renewal; and coverage expansions such as Medicaid expansion under the ACA (Andraka-Christou et al., 2022, Griffin et al., 2023, Haffajee et al., 2018, Harrison et al., 2022, Stein et al., 2023).

Despite substantial growth in the number of waivered clinicians able to prescribe buprenorphine (McBain et al., 2020, Saloner et al., 2021) and the X-waiver’s January 2023 repeal (Bilden et al., 2024, Chua et al., 2024), the buprenorphine treatment gap remains. A majority of clinicians who begin prescribing buprenorphine cease doing so within a year (Cabreros et al., 2021) and many others who obtained X-waivers never prescribed buprenorphine to patients (Duncan et al., 2020, Thomas et al., 2017). Notably, recent studies have demonstrated that a small percentage of high-volume prescribers account for the majority of buprenorphine prescribing (Cabreros et al., 2021, Schuler et al., 2024, Stein et al., 2021) – e.g., over 80 % of all dispensed buprenorphine in 2018 came from the top 17 % of prescribers (Schuler et al., 2024). Despite growing recognition of the significant role played by buprenorphine high-volume prescribers (Stein et al., 2021), few studies have examined how state policies may impact high-volume prescribing. In this paper, we leverage ten years of national pharmacy claims of dispensed buprenorphine prescriptions to examine how state-level policies associated with overall buprenorphine prescribing may specifically relate to high-volume buprenorphine prescribing. Understanding these associations is crucial because shifting the behavior of the relatively few high-volume prescribers may have outsized implications for buprenorphine access and public health.

## 2 Methods

### 2.1 Study population and measures

We identified dispensed buprenorphine prescriptions with an FDA indication for OUD treatment using 2009–2018 deidentified pharmacy claims from IQVIA Real World Data – Longitudinal Prescriptions, which capture an estimated 90 % of all prescriptions filled at U.S. retail pharmacies (IQVIA). These data do not include prescriptions dispensed through the Veterans Health Administration or other non-retail dispensing sites (e.g., inpatient hospitals, opioid treatment programs, or residential treatment facilities). We excluded buprenorphine formulations with indications only for the treatment of pain. For each calendar year, we identified active buprenorphine prescribers as clinicians who wrote a buprenorphine prescription dispensed to at least one patient during that year. For these prescribers, we determined the number of active buprenorphine patients, defined as individuals who received at least one day’s supply of buprenorphine in that month from a dispensed prescription from that clinician. Individuals could be classified as active patients for multiple clinicians within the same month. For each year, we defined *high-volume prescribers* as those who managed an average of 30 or more active buprenorphine patients per month, the patient threshold that determined federal training requirements during our study period (i.e., the initial X-waiver patient limit under DATA 2000). We defined *low-volume prescribers* as those who maintained an average of fewer than 30 active patients during the months in which they were an active prescriber*.* The threshold of 30 active patients, which has historical policy relevance and has been used in prior work (Schuler et al., 2024), corresponded to approximately the 96th percentile of monthly caseloads in our data, capturing prescribers with the largest and most sustained treatment panels.

Using information in the IQVIA data generated from the American Medical Association (AMA) Masterfile (a comprehensive national database of physician demographics, education, and specialty) and refined using the National Plan and Provider Enumeration System (NPPES), which assigns and maintains National Provider Identifiers (NPIs) for U.S. clinicians, we classified prescriber specialty (Centers for Medicare & Medicaid Services). Prescribers were categorized as addiction specialists, including addiction medicine and addiction psychiatry physicians; adult primary care physicians (PCPs), including non-subspecialty internists and family practice physicians; psychiatrists; pain specialists, including anesthesiologists and neurologists; pediatricians; advance practice providers (APPs), including nurse practitioners and physician assistants; emergency medicine specialists; and other prescribers, comprising primarily surgeons and adult subspecialties. Prescriber location was identified using the 5-digit FIPS code in the IQVIA data and classified as urban (RUCC codes 1–3) or rural (RUCC codes 4–9) according to the U.S. Department of Agriculture’s Rural–Urban Continuum Codes (RUCC), a county-level scheme that distinguishes metropolitan from non-metropolitan areas by population size and proximity to urban centers (US Department of Agriculture, E. R. S. 2023). RUCC values were obtained from the Area Health Resources Files (AHRF). County fatal drug overdose rate was calculated as the per capita rate of overdose deaths due to any drug, using the 2015 restricted multiple-cause-of-death mortality file from the Centers for Disease Control and Prevention and classified into quartiles (1 =lowest, 4 =highest) (Centers for Disease Control Prevention, 2020). We additionally controlled for the percentage of Black and Hispanic county residents (classified into quartiles), based on data from the 2015 American Community Survey. The study was approved with a waiver of consent by the RAND Institutional Review Board.

### 2.2 Statistical analysis

Our analysis focused on four state-level policies that prior studies found to be associated with buprenorphine prescribing or waivered prescribers: a) mandatory PDMP laws that require prescribers in a state to check the PDMP before prescribing restricted substances for a patient (RAND-USC Schaeffer OPTIC Center, 2024); b) Medicaid expansion, defined as states that expanded Medicaid coverage under the Affordable Care Act (source: Kaiser Family Foundation); c) CME related to substance misuse and addiction required by states for physician licensure (source: Westlaw database search (Davis et al., 2024)); and d) mandated behavioral health counseling for patients receiving buprenorphine (source: Westlaw database search (Andraka-Christou et al., 2022)).

Our unit of analysis was the clinician-year (2009–2018), and the binary outcome was whether an active buprenorphine prescriber qualified as “high-volume” in that calendar year. To estimate policy associations, we specified a multivariable event-time linear probability model. We used a linear probability model because it generates directly interpretable policy effects estimates in terms of percentage-point changes and easily accommodates multiple fixed effects (Lee et al., 2025). Our primary model specification included all four policies simultaneously, and we examined the effect of the policy for up to three years following implementation. Timing of policy implementation varied across states. We used an event-time framework to assess whether associations between state policies and likelihood of being a high-volume prescriber varied across time after policy implementation (Borusyak et al., 2024, Goodman-Bacon, 2021, Sun and Abraham, 2021). For each policy and state, time was coded as 12-month intervals relative to policy implementation, with negative values for pre-policy periods and positive values for post-policy periods. To select the pre-policy reference year, we examined estimates for potential anticipatory effects. If the estimate at one year before adoption suggested an anticipatory change, we used two years before adoption as the reference category (as was the case for behavioral health counseling and PDMP mandates). When no anticipatory effect was detected, one year before adoption served as the reference (as for CME requirements and Medicaid expansion). We controlled for clinician specialty and county characteristics potentially associated with likelihood of high-volume prescribing, including county rurality, percentage of Black and Hispanic residents, and 1-year lagged fatal overdose rate. We also included state and calendar year fixed effects as well as interaction terms between calendar time fixed effects and a binary indicator for whether a state ever adopted the given policy, allowing us to account for different secular trends across policy and non-policy states. We report estimates as percentage-point changes in the share of high-volume buprenorphine prescribers for years 1, 2, and 3 following implementation of each policy, relative to the pre-policy reference year. Standard error terms were clustered at the state level. We report time-specific estimates and 95 % CIs for each policy of interest. Statistical analysis was performed using SAS, version 9.4 software (SAS Institute Inc).

## 3 Results

We identified 109,387 clinicians who prescribed dispensed buprenorphine from 2009 to 2018 (Table 1). Of these, 8.8 % (n = 9590) were high-volume buprenorphine prescribers in at least one year. The majority of both high-volume and low-volume prescribers were located in urban counties and in counties with higher percentages of Black and Hispanic residents. High-volume prescribers were significantly more likely than low-volume prescribers to be located in counties in the highest quartile of fatal overdose rates. In terms of clinician specialty, a greater proportion of high-volume than low-volume prescribers were psychiatrists (18.5 % vs 11.1 %), addiction specialists (3.8 % vs 0.5 %), and pain specialists (8.6 % vs 7.0 %); adult PCPs comprised the majority of both high-volume (53.8 %) and low-volume (50.6 %) buprenorphine prescribers.

**Table 1:** Characteristics of high volume and non-high volume prescribers, 2009–2018.

*Characteristics*	**High-volume provider**		**Low-volume provider**		Chi-square test
	N = 9590 (8.8 %)		N = 99,797 (91.2 %)		
	N	col%	N	col%	p value
Specialty					< .0001
PCP adult	5155	53.8 %	50,464	50.6 %	
Psychiatrist	1775	18.5 %	11,058	11.1 %	
Addiction specialists	360	3.8 %	535	0.5 %	
Pain specialist	826	8.6 %	6957	7.0 %	
APP	698	7.3 %	9833	9.9 %	
Pediatrician	246	2.6 %	3648	3.7 %	
Emergency physician	208	2.2 %	4753	4.8 %	
Other	322	3.4 %	12,549	12.6 %	
County urbanicity					< .0001
Urban	8336	86.9 %	88,714	88.9 %	
Rural	1254	13.1 %	11,083	11.1 %	
% Black and Hispanic residents 2015 quartile					< .0001
Low (1)	817	8.5 %	4904	4.9 %	
2	1578	16.5 %	11,822	11.8 %	
3	3330	34.7 %	30,194	30.3 %	
High (4)	3865	40.3 %	52,877	53.0 %	
Fatal overdose rate 2015 quartile					< .0001
Low (1)	71	0.7 %	982	1.0 %	
2	1660	17.3 %	27,885	27.9 %	
3	3184	33.2 %	37,882	38.0 %	
High (4)	4675	48.7 %	33,048	33.1 %	
BH counseling required state					< .0001
No	7353	76.7 %	82,222	82.4 %	
Yes	2237	23.3 %	17,575	17.6 %	
Mandatory CME state					< .0001
No	3198	33.3 %	35,534	35.6 %	
Yes	6392	66.7 %	64,263	64.4 %	
Medicaid expansion state					< .0001
No	2570	26.8 %	29,738	29.8 %	
Yes	7020	73.2 %	70,059	70.2 %	
Mandatory PDMP states					< .0001
No	4295	44.8 %	58,648	58.8 %	
Yes	5295	55.2 %	41,149	41.2 %	

**Note:** In this table, the high-volume group comprises prescribers who met high-volume criteria for at least one 12-month period in the study period.

We further characterized the distribution of caseloads for high-volume and low-volume prescribers (Appendix Table). Among low-volume prescribers, median caseloads were typically just one active patient, and average caseloads ranged from about three to five patients across study years. In contrast, high-volume prescribers maintained much larger patient panels, with median caseloads of approximately 50–65 patients, means of 60–80, and 95th percentiles exceeding 120. These distributions highlight the pronounced divide between the two groups: most low-volume prescribers treated only one or two patients, whereas high-volume prescribers managed substantially greater patient volumes.

**Fig. 1:**
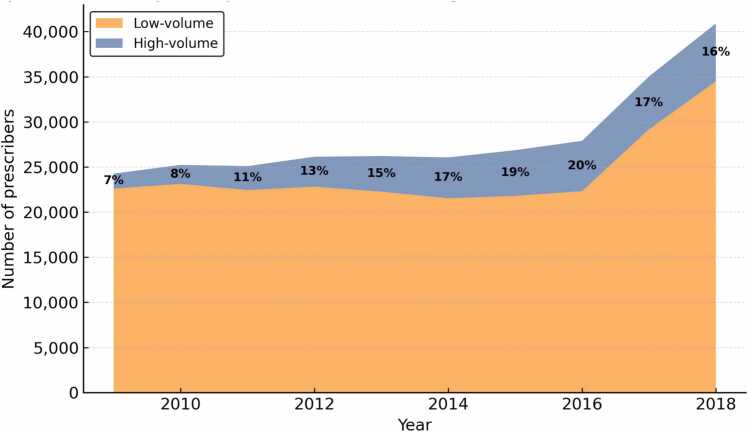
Trends in High-Volume and Low-Volume (non-HV) Buprenorphine Prescribers, 2009–2018. **Note:** Figure displays the number of active buprenorphine prescribers per year, stratified by high-volume (30 + average active patients/month) and low-volume (<30 patients/month). Percentages represent the share of high-volume prescribers among all active prescribers in each year.

Fig. 1 presents trends in the absolute number of high- and low-volume buprenorphine prescribers from 2009 to 2018. The overall number of active prescribers grew substantially, from 24,263 in 2009–40,897 in 2018. Both groups expanded: high-volume prescribers increased from 1663 to 6408, while low-volume prescribers rose from 22,600 to 34,489. The notable increase in low-volume prescribers beginning in 2016 likely reflects the Comprehensive Addiction and Recovery Act (CARA), which expanded prescribing privileges to advanced practice providers (APPs) such as nurse practitioners and physician assistants. At the same time, high-volume prescribers also grew rapidly, with the proportion of high-volume prescribers rising from 7 % in 2009–16 % in 2018. These patterns indicate that the rising proportion of high-volume prescribers was not driven by attrition among lower-volume prescribers; rather, both groups expanded, with faster growth among high-volume prescribers within a steadily increasing overall workforce.

**Fig. 2:**
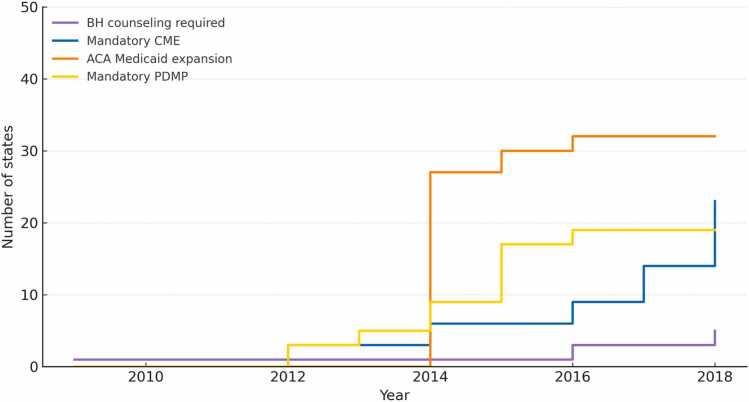
Year of state policy implementation, January 2009 to December 2018. **Note:** Policies shown include: (1) Behavioral Health (BH) Counseling – state laws requiring patients prescribed buprenorphine to receive behavioral health counseling; (2) Continuing Medical Education (CME) – state requirements for substance use disorder–related CME for clinician licensure; (3) ACA Medicaid Expansion – state adoption of Medicaid coverage expansion under the Affordable Care Act; and (4) Mandatory Prescription Drug Monitoring Program (PDMP) – state laws requiring prescribers to check the PDMP before prescribing controlled substances.

**Fig. 3:**
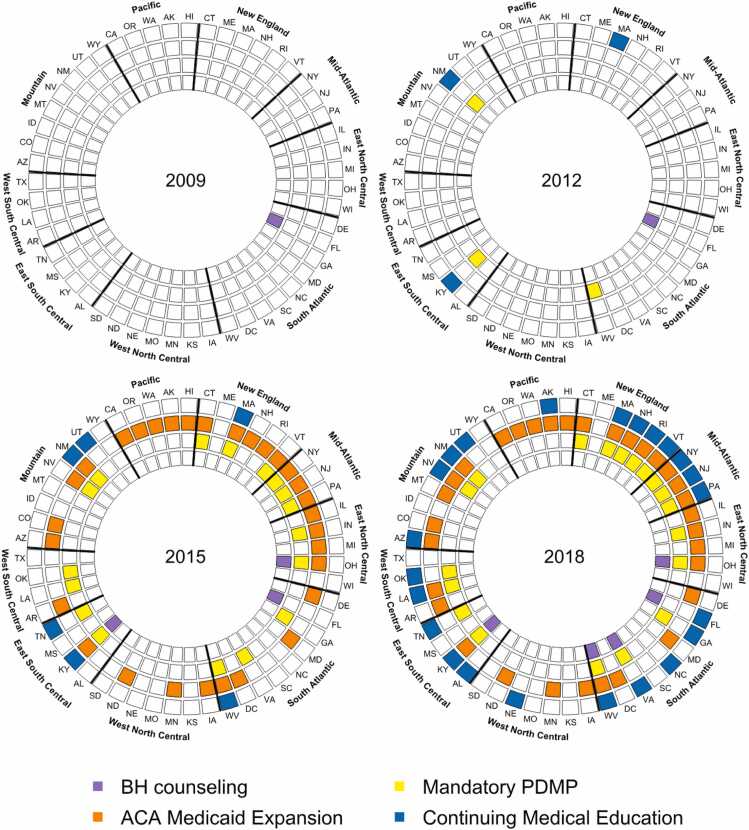
Concurrent policy adoptions by state in selected years. **Note:** Selected years illustrate staggered state adoption of the four policies. States may implement multiple policies concurrently.

Fig. 2, Fig. 3 illustrate the staggered adoption of the four policies of interest between 2009 and 2018. By December 2018, 32 states had expanded Medicaid, 23 had CME requirements regarding substance use disorders, 19 had mandatory PDMP laws, and 5 mandated behavioral health counseling for buprenorphine patients (Fig. 2). Fig. 3 illustrates the growing concurrent implementation of these policies – by 2018, 14 states had implemented at least three of these four policies.

**Fig. 4:**
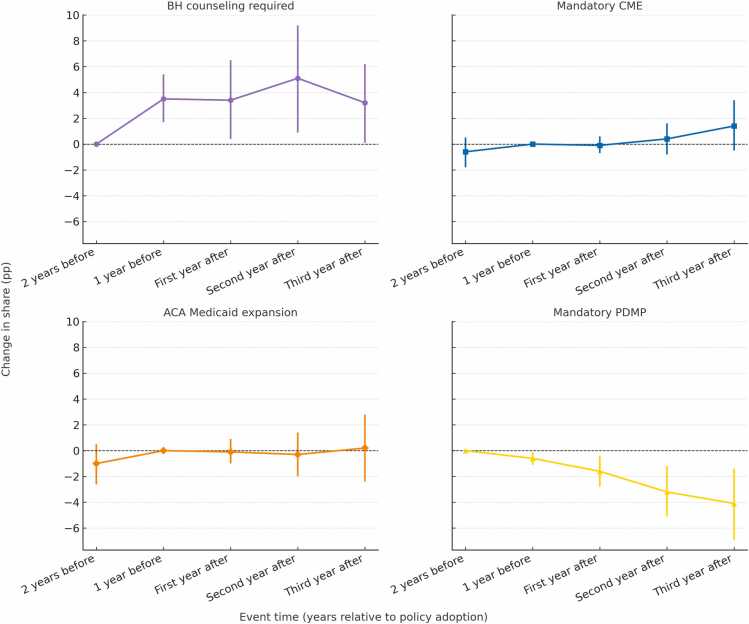
Annual change (percentage points) in share of high-volume buprenorphine providers within 3 years following implementation of four state policies of interest. Note: Estimates are expressed relative to the pre-policy reference year (effect estimate = 0). Model controlled for clinician specialty, county rurality, county percentage of Black and Hispanic residents, and 1-year lagged fatal county overdose rate, as well as state and calendar year fixed effects. Interaction terms between calendar year fixed effects and indicators for whether a state ever adopted each policy were included to account for differential secular trends.

Table 2 and Fig. 4 present the event-time estimates for each policy, based on a regression model that controls for county characteristics and all policies examined. Estimates represent changes relative to the selected pre-policy reference year (one year prior to adoption unless anticipatory effects were detected, in which case two years prior was used). Our findings indicate that state policies mandating behavioral health counseling for patients prescribed buprenorphine were associated with a significant increase in the proportion of active buprenorphine prescribers classified as high-volume in the three years following policy implementation. Specifically, the proportion of high-volume prescribers increased 3.4 %age points (95 % CI = 0.4, 6.5) in the first year after implementation, 5.1 %age points (95 % CI = 0.9, 9.2) in the second year, and 3.2 %age points (95 % CI = 0.1, 6.2) in the third year.

**Table 2:** Annual change (percentage points) in share of high-volume buprenorphine providers within 3 years following implementation of four state policies of interest.

	Effect (95 % CI)			
Time Point	BH counseling required	Mandatory CME	ACA Medicaid expansion	Mandatory PDMP
2 years before adoption	*Ref*	-0.6 (-1.8, 0.5)	-1 (-2.6, 0.5)	*Ref*
1 year before adoption	**3.5 (1.7, 5.4)**	*Ref*	*Ref*	**-0.6 (-1.1, −0.1)**
First year after	**3.4 (0.4, 6.5)**	-0.1 (-0.7, 0.6)	-0.1 (-1, 0.9)	**-1.6 (-2.8, −0.4)**
Second year after	**5.1 (0.9, 9.2)**	0.4 (-0.8, 1.6)	-0.3 (-2, 1.4)	**-3.2 (-5.1, −1.2)**
Third year after	**3.2 (0.1, 6.2)**	1.4 (-0.5, 3.4)	0.2 (-2.4, 2.8)	**-4.1 (-6.9, −1.4)**

**Note:** Bold denotes estimates significant at the 0.05 level. Estimates are expressed relative to the stated pre-policy reference year. Model controlled for clinician specialty, county rurality, county percentage of Black and Hispanic residents, and 1-year lagged fatal county overdose rate, as well as state and calendar year fixed effects. Interaction terms between calendar year fixed effects and indicators for whether a state ever adopted each policy were included to account for differential secular trends.

Conversely, we found that mandatory PDMP laws were associated with a significant decrease in the proportion of active buprenorphine prescribers who were high-volume prescribers in the three years following policy implementation. We observed a decrease of 1.6 %age point (95 % CI = −2.8, −0.4) in high-volume prescribers in the first year after implementation, a 3.2 %age point decrease (95 % CI = −5.1, −1.2) in the second year, and a decrease of 4.1 %age points (95 % CI = −6.9, −1.4) in the third year.

There was no significant association between high-volume buprenorphine prescribing and the implementation of mandatory continuing medical education (CME) requirements for buprenorphine prescribers or the expansion of Medicaid under the Affordable Care Act.

## 4 Discussion

In this national study of over 109,000 buprenorphine providers from 2009 to 2018, we found that state policies mandating behavioral health counseling for individuals receiving buprenorphine were associated with significant increases in the proportion of high-volume prescribers. As other recent studies using national IQVIA prescribing data have found, high-volume prescribers increasingly play a very central role in providing MOUD (Cabreros et al., 2021, Schuler et al., 2024, Stein et al., 2021). Because such a small share of prescribers drives overall treatment availability, policies that affect high-volume prescribers may have a particularly sizable impact on buprenorphine access. In contrast, we observed no evidence that mandatory CME requirements or Medicaid expansion were associated with changes in high-volume prescribing. These findings suggest that state policies may differentially shape buprenorphine workforce composition, with some policies coinciding with expansion of high-volume treatment capacity and others with contraction.

The association between behavioral health counseling mandates and growth in high-volume prescribers is particularly noteworthy. While assessing the need for behavioral health referral has long been a standard component of OUD care, clinicians often report trepidation about managing patients with OUD and cite lack of access to behavioral health and substance use treatment services for patients as key barriers to prescribing buprenorphine (Gordon et al., 2011, Hutchinson et al., 2014, Winograd et al., 2023). On face value, unfunded counseling mandates may seem unlikely to increase provider comfort with buprenorphine treatment, particularly in settings where behavioral health resources are scarce. However, by codifying counseling requirements into law, states may signal stronger support for integrated treatment and reduce clinicians’ sense of being solely responsible for OUD care, which may, in part, explain why clinicians under these mandates appear more willing to manage larger buprenorphine caseloads. It is possible that behavioral-health-counseling mandates may pose barriers for early or low-volume prescribers with limited infrastructure for OUD treatment, who may find establishing referral processes daunting, while reinforcing engagement among more experienced prescribers who already have such systems in place. Overall, as the availability of behavioral health services remains limited, the effectiveness of such mandates likely depends on parallel investments in behavioral health infrastructure.

Conversely, we found that implementation of mandatory prescription drug monitoring programs (PDMPs) laws, which require clinicians to check the state PDMP before prescribing buprenorphine, was associated with a significant decrease in the proportion of high-volume buprenorphine prescribers. PDMPs were established to monitor and curb high-volume opioid analgesic prescribing, and their deterrent effects can play a key role in reducing clinically unnecessary opioid use. However, while checking a PDMP may take little time, the requirement can disrupt clinical workflow and has been shown to influence prescribing behavior (Picco et al., 2021, Tobin, 2018, Wen et al., 2019). As such, PDMP mandates may inadvertently decrease buprenorphine prescribing, including among high-volume prescribers. Notably, a study of PDMP use in Texas prior to mandated use found that clinicians were more likely to query buprenorphine prescriptions than other opioids, suggesting that heightened scrutiny or stigma toward patients receiving OUD treatment may drive this behavior (Thornton et al., 2021). Yet PDMPs provide limited information relevant to buprenorphine initiation because they do not capture illicit substance use or diverted buprenorphine, and most clinicians receive little training on how to interpret PDMP data. These dynamics highlight a critical tension in policy design: while aiming to prevent opioid analgesic misuse, policymakers must also consider the potential negative impact on MOUD treatment availability.

These policy-related changes occurred against a backdrop of substantial overall workforce expansion. The number of active buprenorphine prescribers rose from just over 24,000 in 2009 to nearly 41,000 in 2018, with both high- and low-volume prescribers increasing over time. Across the study period, median caseloads highlight a stark divide: low-volume prescribers typically treated only one or two patients, consistent with prior characterizations of “dabbling” behavior, whereas high-volume prescribers sustained panels of roughly 50–65 patients. The share of prescribers classified as high-volume nearly doubled, from 7 % to 16 %, reflecting faster growth among this small but critical subset. High-volume prescribers were more likely than low-volume prescribers to be located in urban counties, in counties with higher proportions of Black and Hispanic residents, and in areas with high overdose rates, patterns consistent with prior work showing that buprenorphine capacity tends to cluster in urban and more racially diverse communities disproportionately affected by the opioid crisis (Stein et al., 2021, Schuler et al., 2021). Notably, the sharp increase in prescriber counts beginning in 2016 coincides with the Comprehensive Addiction and Recovery Act, which extended prescribing privileges to advanced practice providers; by 2018, APPs accounted for nearly 3 % of high-volume prescribers.

Although requiring continuing medical education (CME) for buprenorphine prescribers has been associated with an increase in buprenorphine prescribing per capita (Stein et al., 2023), we did not find a significant association with high-volume prescribing. The association with increased per capita buprenorphine prescribing previously observed may reflect an association with an increase in low-volume prescribing. Most low-volume buprenorphine prescribers are adult PCPs, and prior studies have found that non-specialist clinicians often raise concerns that patients with OUD are clinically complex, citing their lack of knowledge and training on OUD treatment and buprenorphine as barriers to buprenorphine prescribing (Foti et al., 2021, Jones and McCance-Katz, 2019, Mackey et al., 2020, Winograd et al., 2023). Such barriers may be less common in clinicians such as addiction specialists and psychiatrists, whose training likely included treatment of these complex patients. Thus, mandated CME may be more likely to stimulate changes in prescribing behavior among non-specialist clinicians than among clinicians already comfortable prescribing buprenorphine to greater numbers of patients. We also found no evidence of an association between Medicaid expansion and high-volume prescribing, consistent with prior studies examining state policies and overall buprenorphine prescribing per capita (Stein et al., 2023).

Our findings must be considered in the context of study limitations. The IQVIA data used in this study reflect an estimated 90 % of prescriptions dispensed at U.S. retail pharmacies but may not fully generalize to dispensing settings not captured in IQVIA, including VA pharmacies, inpatient hospitals, opioid treatment programs, and residential treatment settings. We have no information on the race/ethnicity or clinical status of patients (e.g., diagnoses and clinical complexity) to whom buprenorphine was dispensed, nor information on prescriber practice setting--all factors that can potentially influence buprenorphine prescribing. We do not have more recent buprenorphine pharmacy data and are unable to examine how more recent policy changes, such as the abolition of the X-waiver, might influence our findings. Analyses were restricted to buprenorphine formulations indicated for OUD treatment but may include claims that represent off-label use of those formulations. Our definition of high-volume prescribers (≥30 active patients per month during months when a clinician was actively prescribing) may classify some who briefly exceeded this threshold and then stopped prescribing as high-volume for the year. However, prior longitudinal work suggests that attrition is concentrated among low-volume prescribers, and it is rare for high-volume prescribers to cease prescribing altogether (Cabreros et al., 2021). Thus, any resulting misclassification is likely minimal and would bias estimated policy associations toward the null. The associations observed in our study may, in part, reflect the impact of other opioid-related policies or other exogenous factors at the state level. For example, we are not able to account for regulations or policies occurring at the local, regional, or health system (e.g., Veterans Health Administration) level.

## 5 Conclusion

Despite limitations, this study contributes to the growing literature on high-volume buprenorphine prescribers and the role of state policies in shaping treatment capacity. High-volume prescribers represent a small share of all buprenorphine providers but account for most dispensed prescriptions, and within our dataset (2009–2018), their proportion more than doubled, underscoring their central role in expanding MOUD access. We found that state policies requiring behavioral health counseling for patients prescribed buprenorphine were associated with significant increases in the proportion of high-volume buprenorphine prescribers, whereas mandatory PDMP laws were associated with significant decreases. As states continue to respond to high rates of opioid overdose, understanding how policies influence the composition of the buprenorphine workforce, particularly the subset of high-volume prescribers, will be critical for efforts to expand equitable access to effective OUD treatment.

## Contributors

MSS, AJG, BS, and BDS conceptualized the study. FS performed analyses. MSS led manuscript writing. AJG, BS, and BDS contributed to interpretation of results, manuscript writing, and revision. BDS obtained funding and acquisition of study data. All authors have read and approved the final version of the manuscript. The authors thank Hilary Peterson and Mary Vaiana of RAND for their feedback and editorial assistance on earlier versions of the manuscript.

## CRediT authorship contribution statement

**Flora Sheng:** Visualization, Methodology, Formal analysis, Data curation. **Brendan Saloner:** Writing – review & editing, Methodology, Conceptualization. **Megan S. Schuler:** Writing – review & editing, Writing – original draft, Methodology, Investigation, Conceptualization. **Adam J. Gordon:** Writing – review & editing, Methodology, Conceptualization. **Bradley D. Stein:** Writing – review & editing, Supervision, Resources, Methodology, Funding acquisition, Conceptualization.

## Role of funding source

This work was funded by 10.13039/100000026NIDA awards R01 DA045800 and P50 DA046351. The content is solely the responsibility of the authors and does not necessarily represent the official views of NIDA, the 10.13039/100000002NIH or the US Government.

## Declaration of Competing Interest

None

## Figures and Tables

**Table A1:** Caseload distributions among low-volume and high-volume buprenorphine prescribers, 2009–2018

Year	Group	# Providers	Mean	5th Pctl	10th Pctl	25th Pctl	Median	75th Pctl	90th Pctl	95th Pctl	Max
2009	Low-volume	22,600	4	1	1	1	1	3	11	18	30
	High-volume	1663	61	31	33	38	50	71	97	127	430
2010	Low-volume	23,122	4	1	1	1	1	3	12	18	30
	High-volume	2111	59	31	33	38	50	68	93	119	490
2011	Low-volume	22,437	4	1	1	1	1	3	12	19	30
	High-volume	2660	62	32	33	39	53	72	95	123	487
2012	Low-volume	22,819	4	1	1	1	1	4	13	20	30
	High-volume	3316	65	32	34	41	56	76	100	129	489
2013	Low-volume	22,261	4	1	1	1	1	4	14	20	30
	High-volume	3952	68	32	35	43	59	78	106	134	468
2014	Low-volume	21,527	5	1	1	1	1	5	15	21	30
	High-volume	4532	69	32	35	43	60	80	108	139	532
2015	Low-volume	21,791	5	1	1	1	1	5	16	22	30
	High-volume	5067	70	32	35	45	62	82	109	136	677
2016	Low-volume	22,316	5	1	1	1	1	5	16	22	30
	High-volume	5575	71	32	35	44	62	83	111	143	536
2017	Low-volume	29,159	5	1	1	1	1	5	15	21	30
	High-volume	5865	77	32	35	44	64	93	135	174	443
2018	Low-volume	34,489	5	1	1	1	2	6	15	21	30
	High-volume	6408	80	32	35	43	63	96	149	191	654

**Note:** Low-volume prescribers are those with < 30 active buprenorphine patients per month; high-volume prescribers are those with ≥ 30. Values represent the distribution of average monthly patient caseloads during months in which a prescriber was active. All values are rounded to whole numbers.
